# Prognostic role of inflammatory and tumor biomarkers in hilar cholangiocarcinoma patients receiving postoperative adjuvant therapy

**DOI:** 10.3389/fonc.2025.1555369

**Published:** 2025-04-24

**Authors:** Di Zeng, Saud Ahmad Saad, Zhen You, Nansheng Cheng

**Affiliations:** ^1^ Division of Biliary Tract Surgery, Department of General Surgery, West China Hospital, Sichuan University, Chengdu, Sichuan, China; ^2^ Research Center for Biliary Diseases, West China Hospital, Sichuan University, Chengdu, Sichuan, China

**Keywords:** hilar cholangiocarcinoma, systemic immune-inflammation index, adjuvant therapy, cancer prognosis, tumor biomarker

## Abstract

**Background:**

Hilar cholangiocarcinoma (HCCA) is an aggressive cancer with poor prognosis after surgery. The systemic immune-inflammation index (SII) has been proposed as a prognostic marker, but its relationship with other markers such as CA19-9 remains unclear. This study investigates the prognostic significance of SII and CA19-9 in HCCA patients receiving post-surgery adjuvant therapy.

**Methods:**

A cohort of 145 HCCA patients who underwent surgery and adjuvant therapy was analyzed. Patients were categorized into High SII and Low SII groups based on an optimal cutoff value of 672.8, determined using ROC curve analysis. Further stratification was performed based on CA19-9 levels. The associations between SII, CA19-9, and survival outcomes, including overall survival (OS) and disease-free survival (DFS), were assessed using Kaplan-Meier survival analysis and Cox proportional hazards regression.

**Results:**

Elevated SII was significantly associated with worse OS (p = 0.0027) and DFS (p = 0.0024). Notably, a significant difference in CA19-9 levels was observed between high and low SII groups (p = 0.013), with higher CA19-9 levels in the high SII group. However, no significant difference in CA19-9 was found between the low SII groups (p = 0.128). Patients with both high SII and high CA19-9 levels had the poorest survival outcomes, with significantly higher risks of mortality and disease recurrence (HR for OS = 2.29, 95% CI: 1.23–4.25; HR for DFS = 2.16, 95% CI: 1.17–3.99). Multivariate analysis identified high SII, high CA19-9, lymph node metastasis, and local organ metastasis as independent prognostic factors.

**Conclusions:**

Elevated SII and CA19-9 are independent prognostic markers for HCCA patients after surgery. The combination of high SII and high CA19-9 identifies a subgroup with the poorest prognosis, suggesting the potential for these markers to guide postoperative treatment decisions.

## 1 Introduction

Hilar cholangiocarcinoma (HCCA) is a highly aggressive malignancy originating from the bile ducts at the hepatic hilum, characterized by a poor prognosis and high recurrence rates (1). For hilar cholangiocarcinoma (HCCA), surgical resection remains the only curative treatment (2). However, even after curative-intent surgery, the 5-year overall survival (OS) rate is unsatisfactory, ranging from 25% to 40%, owing to high recurrence and metastasis rates (3). Adjuvant therapy, including chemotherapy and radiotherapy, is frequently used after surgery to reduce recurrence and improve survival (4). However, the efficacy of adjuvant therapy is highly variable, and factors influencing the response to treatment are still not fully understood.

The Systemic Immune-Inflammation Index (SII), a marker that combines the platelet, neutrophil, and lymphocyte counts, provides a comprehensive measure of systemic inflammation and immune status (5). The SII reflects the balance between pro-tumor inflammation and anti-tumor immunity. Neutrophils support tumor progression by promoting angiogenesis, immune evasion, and metastasis, while platelets shield tumor cells and aid in pre-metastatic niche formation (6). Conversely, lymphocytes play a key role in anti-tumor immunity by targeting tumor cells. A high SII, marked by elevated neutrophils and platelets and reduced lymphocytes, indicates an immunosuppressive environment that fosters tumor growth and spread (7). Increased levels of SII have been associated with worse prognosis in various malignancies, including hepatocellular carcinoma (8), colorectal cancer (9), gastric cancer (10), and pancreatic cancer (11), where it reflects an immunosuppressive microenvironment that favors tumor growth and metastasis.

The prognostic significance of the Systemic Immune-Inflammation Index (SII) extends to its ability to predict responses to adjuvant therapies across various cancers. Elevated SII levels have been consistently associated with lower efficacy of adjuvant chemotherapy and radiotherapy (12), likely due to the immunosuppressive microenvironment it reflects, which impairs the immune system’s tumor-killing ability. For example, in non-small cell lung cancer, patients with high SII levels demonstrate worse responses to postoperative adjuvant chemotherapy, potentially due to systemic inflammation reducing treatment effectiveness (13). Similarly, in breast cancer, high SII predicts poorer outcomes for patients receiving adjuvant chemotherapy (14). These findings highlight the potential of SII as a predictive biomarker for stratifying patients and tailoring individualized adjuvant treatment strategies. Incorporating SII into prognostic models may further refine therapy selection, optimizing outcomes for cancer patients (15).

However, the specific role of SII in HCCA patients undergoing adjuvant therapy remains unclear. Given HCCA’s unique biological characteristics and treatment variability, combining SII with other biomarkers, such as carbohydrate antigen 19-9 (CA19-9), may enhance its predictive and prognostic value (16). Understanding how SII interacts with immune responses and CA19-9 levels in HCCA could offer valuable insights into optimizing adjuvant therapy and improving survival outcomes. While CA19-9 reflects tumor burden and biological aggressiveness, SII represents systemic inflammation and immune status, suggesting that their integration could provide a more comprehensive evaluation of patient prognosis and guide personalized treatment strategies. Exploring such combinations may not only enhance risk stratification but also improve therapeutic decision-making, paving the way for more personalized treatment strategies in HCCA.

## 2 Methods

### 2.1 Patients

We retrospectively collected data from patients with hepatocellular carcinoma (HCC) who underwent curative-intent resection and received postoperative adjuvant therapy at West China Hospital, Sichuan University, Chengdu, China, between January 2014 and December 2024. A total of 145 patients were included in this study. The study was approved by the Ethics Committee of West China Hospital (approval No. 2022-1774). All patients signed informed consent forms prior to surgery.

Inclusion Criteria:

Diagnosis of Hilar Cholangiocarcinoma (HCCA): Patients diagnosed with hilar cholangiocarcinoma (HCCA) confirmed by both histopathological and radiological findings; Surgical Resection: Patients who underwent curative-intent surgical resection of HCCA at West China Hospital, Sichuan University, between January 2014 and January 2024, with a minimum follow-up duration of 12 months; Adjuvant Therapy: Patients who received post-operative adjuvant therapy (chemotherapy or radiation therapy); Complete Clinical Data: Availability of complete pre-operative and post-operative clinical data, including laboratory results, imaging, and treatment details; Age: Patients aged 18 years or older; Ethical Approval: Signed informed consent for participation in the study.

Exclusion Criteria:

Missing Data: Patients with missing or incomplete data required for the calculation of the systemic immune-inflammation index (SII) or other key variables, such as survival days or treatment details; Non-curative Resection: Patients who underwent palliative surgery or whose surgery was not intended to achieve curative resection; Other Malignancies: Patients with a history of other malignancies within the past five years, except for non-melanoma skin cancer; Inadequate Follow-Up Data: Patients with inadequate follow-up data or those who did not meet the criteria for survival or free survival analysis.

### 2.2 Study outcomes and definition

The primary outcome of this study was overall survival (OS), defined as the time from the date of surgery to the date of death from any cause during the study period. Patients who were alive at the last follow-up were censored. The secondary outcome was disease-free survival (DFS), defined as the time from the date of surgery to the date of disease recurrence or progression, or the date of death from any cause, whichever occurred first.

The Systemic Immune-Inflammation Index (SII) was calculated based on the formula (8):

***platelet count ×neutrophil count/lymphocyte count and expressed as × 109 cells/μl***

The SII was categorized into low and high groups based on the median value of the cohort, which was consistent with prior studies.

Additionally, the following clinical parameters were assessed for their potential association with survival outcomes:

CA19-9: Serum levels of cancer antigen 199 (CA19-9) were measured preoperatively as a marker for tumor burden. Elevated CA19-9 levels were considered indicative of advanced disease. Blood samples for SII calculation (platelet, neutrophil, and lymphocyte counts) and CA19-9 levels were collected preoperatively within 7 days prior to surgery, before any neoadjuvant therapy or surgical intervention.

Systemic Therapy: Patients received different adjuvant therapies, including chemotherapy and radiation therapy, following curative-intent resection. The type of therapy was recorded as a categorical variable (e.g., chemotherapy, radiotherapy, or a combination of both).

**Figure 1:**
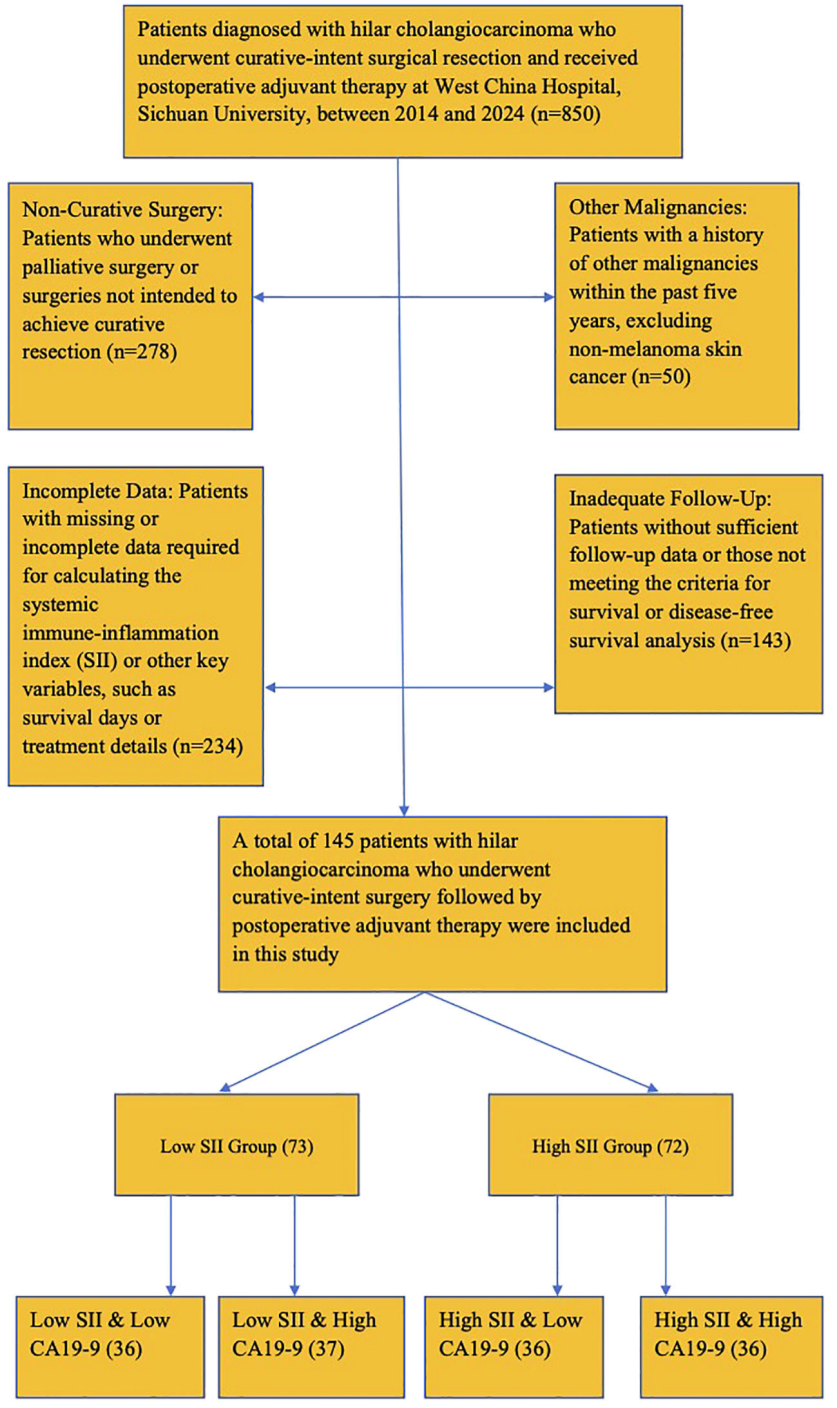
Patient inclusion flow diagram. Flowchart showing the inclusion and exclusion criteria applied to select study participants.

Follow-up was conducted at regular intervals post-surgery, with data recorded at 3-month intervals for the first year and at 6-month intervals thereafter. The final follow-up was conducted in June 2024. The complete patient inclusion process is shown in **Figure 1**.

### 2.3 Data collection

Patient demographic data, disease history, and laboratory test data were collected. The clinical data extracted for this study included systemic immune-inflammation index (SII), calculated as the platelet count × neutrophil count/lymphocyte count, tumor markers (CA19-9, CEA, CA125), and various laboratory results such as hemoglobin, bilirubin (total and direct), albumin, ALT, AST, platelet count, prothrombin time, and Child-Pugh score. Tumor characteristics such as tumor size, type, differentiation, and invasion of adjacent vessels and organs were also recorded. Additionally, postoperative outcomes such as complications including liver failure, bile leakage, and bleeding, hospital mortality, and reoperation rates were documented. The status of lymph node metastasis, local organ metastasis, and intrahepatic metastasis was also included. Patients were followed up regularly after surgery through outpatient visits and/or telephone consultations. The follow-up period lasted from the date of surgery until the last available clinical visit or the occurrence of death, whichever came first. During follow-up, data regarding long-term survival, recurrence, and complications were updated. Follow-up data were collected at intervals of 3, 6, and 12 months post-surgery, with further visits conducted as clinically indicated. Information on survival status (alive or deceased), recurrence of disease, and other postoperative complications was recorded throughout the follow-up period.

### 2.4 Statistical analysis

Patient data were retrospectively collected and analyzed using SPSS version 25.0 (SPSS Inc., Chicago, IL, USA). Continuous variables with a normal distribution are presented as mean ± standard deviation (SD), while non-normally distributed data are expressed as median and range. Categorical variables are reported as absolute values and percentages. For normally distributed continuous data, comparisons between groups were performed using the Student’s t-test, while the Mann-Whitney U-test was applied for data with skewed distributions. The χ2 test or Fisher’s exact test was used for comparing categorical data. Survival analysis was conducted using the Kaplan–Meier method, and differences between subgroups were assessed with the log-rank test. Multivariate analysis for prognostic factors was performed using the Cox proportional hazards model, focusing on variables with P < 0.05 in univariate analysis. The optimal cutoff value for SII was determined using ROC curve analysis based on overall survival (OS) and disease-free survival (DFS). The “surv_cutpoint” function in R was employed to identify the threshold that maximized the log-rank statistic, ensuring the strongest separation between high and low SII groups. This method aligns with established practices in prognostic biomarker research and enhances the biological and clinical relevance of our findings.

A two-sided P value < 0.05 was considered statistically significant. Additionally, subgroup analyses were conducted based on gender, age, TNM staging, Bismuth staging, tumor grade, tumor size, margin status, preoperative bile duct drainage, and extent of tumor invasion. The data for each subgroup were summarized in a forest plot.

## 3 Results

### 3.1 Baseline characteristics of study participants

A total of 145 patients with hilar cholangiocarcinoma (HCCA) who underwent surgery and received post-surgery adjuvant therapy were included in this study. Based on the systemic immune-inflammation index (SII), the patients were divided into two groups: a high SII group (n = 65) and a low SII group (n = 80) based on an optimal cutoff value of 672.8, determined using ROC curve analysis.

The baseline characteristics of the study participants are summarized in **Table 1A**. Most of the baseline characteristics were similar between the two groups, including gender distribution, age, hemoglobin levels, liver function markers (total bilirubin, direct bilirubin, albumin), liver enzymes (ALT, AST), platelet counts, prothrombin time, tumor size, hospital stay, Child-Pugh grade, and tumor differentiation. Tumor characteristics such as the distribution of tumor types, differentiation status, and invasion of adjacent structures (artery, vein, main hepatic vein, vena cava) were also comparable between the two groups. Similarly, the presence of metastasis (local organ, intrahepatic, and lymph node), TNM staging, AJCC stage, jaundice, biliary drainage, and HBV history did not show significant differences. Postoperative outcomes, summarized in **Table 1B**, showed no significant differences between the two groups in terms of hospital mortality (p = 0.93) or reoperation rates (p = 0.99). Complications such as liver failure (Grade A: p = 0.35, Grade B/C: p = 0.99), bile leakage (Grade A: p = 0.98, Grade B/C: p = 0.47), and postoperative bleeding (Grade A: p = 0.99, Grade B/C: p = 0.99) were comparable across the groups. The Clavien-Dindo grade of complications showed no significant difference (p = 0.20), with the majority of patients experiencing Grade I or less complications in both groups. Similarly, no significant differences were observed in the rates of no complications (p = 0.35), 90-day mortality (p = 0.60), or 90-day readmission (p = 1.00). Other factors, including blood transfusion requirements (p = 0.68), R0 resection rates (p = 0.41), and textbook outcome achievement (p = 0.40), were also balanced between the two groups. However, a significant difference was observed in the levels of CA19-9, which were notably higher in the high SII group compared to the low SII group (p = 0.04). Other tumor markers such as CEA and CA125 showed no statistically significant differences between the groups.

**Table 1A:** Demographic and baseline clinical characteristics of study participants.

Variables	SII Low		SII High		p_value
Gender					
Male	43		46		0.66
Female	30		26		
Age	60.26	(53.00, 66.00)	61.29	(57.21, 65.00)	0.25
Hemoglobin (g/L)	125.00	(112–137)	124.00	(110.5-135.25)	0.27
Total Bilirubin (µmol/L)	118.21	(21.1-178.8)	133.82	(37.27-208.82)	0.44
Direct Bilirubin (µmol/L)	98.21	(11.2-156.1)	110.90	(33-182.98)	0.45
Albumin (g/L)	38.73	(35.6-41.6)	38.15	(35.48-40.5)	0.46
ALT (U/L)	97.93	(37-124)	129.58	(46-138.5)	0.11
AST (U/L)	89.61	(39-104)	103.19	(41-130.5)	0.36
Platelet (×10^9/L)	210.67	(140-267)	233.42	(172.75-287.25)	0.13
Serum CEA (ng/mL)	10.62	(2-6.01)	18.86	(2.37-8.99)	0.37
Serum CA 125 (U/mL)	52.96	(16.9-55.3)	57.31	(19.44-77.88)	0.70
Serum CA 19-9 (U/mL)	386.74	(57.62-718.2)	522.17	(85.02-1000)	0.04
Prothrombin Time (seconds)	11.38	(10.4-12.3)	11.57	(10.67-12.12)	0.38
Processed Tumor Size (cm)	4.86	(3.6-5.5)	4.93	(3.4-6)	0.81
Hospital Stay (days)	19.11	(12-22)	17.24	(12-20.25)	0.29
Child_Pugh_Grade					0.24
A	27		21		
B	46		49		
C	0		2		
Jaundice					0.09
Yes	56		8		
No	17		64		
biliary.drainage					0.65
Yes	45		48		
No	28		24		
Tumor.Type					0.28
Adenocarcinoma	71		67		
Adenosquamous carcinoma	1		1		
Mucinous adenocarcinoma	1		4		
Tumor Differentiation					0.38
Well-differentiated	0		0		
Moderately differentiated	4		4		
Moderately to poorly differentiated	50		56		
Poorly differentiated	19		12		
Artery.invasion					0.77
Yes	25		22		
No	48		50		
Vein.invasion					0.61
Yes	21		17		
No	52		55		
Main.hepatic.vein.invasion					0.42
Yes	16		21		
No	57		51		
Vena.cava.invasion					0.28
Yes	6		2		
No	67		70		
Local.organ.metastasis					0.23
Yes	4		9		
No	69		63		
Intrahepatic.metastasis					0.53
Yes	15		19		
No	58		53		
Lymph.node.metastasis					0.21
Yes	28		36		
No	45		36		
T STAGE					0.17
I	11		12		
II	21		28		
III	36		23		
IV	5		9		
N STAGE					0.36
N0	45		38		
N1	28		34		
M STAGE					0.93
M0	73		72		
M1	0		0		
AJCC STAGE					0.16
IA	6		9		
II	16		19		
IIIA	30		17		
IIIB	21		27		
HBV History					0.66
Yes	15		18		
No	58		54		
Adjuvant Therapy					
Gemcitabine + Capecitabine	30		33		
Gemcitabine + Cisplatin	27		29		
Tegio Oral	10		9		
Capecitabine Oral	2		1		
FOLFIRINOX	2		0		
GEMOX	2		0		

Baseline characteristics of patients with hilar cholangiocarcinoma stratified by systemic immune-inflammation index (SII).

**Table 1B:** Postoperative outcomes and complications.

Variables	SII Low	SII High	p_value
Hospital.mortality			0.93
Yes	0	0	
No	73	72	
Reoperation			0.99
Yes	2	3	
No	71	69	
Grade.A.liver.failure			0.35
Yes	1	4	
No	72	68	
Grade.B.C.liver.failure			0.99
Yes	1	2	
No	72	70	
Grade.A.bile.leakage			0.98
Yes	6	7	
No	67	65	
Grade.B.C.bile.leakage			0.47
Yes	0	2	
No	73	70	
Grade.A.postoperative.bleeding			0.99
Yes	2	3	
No	71	69	
Grade.B.C.postoperative.bleeding			0.99
Yes	1	1	
No	72	71	
Clavien.Dindo.grade			0.20
Grade I or Less	38	43	
Grade II	12	4	
Grade IIIa	15	12	
Grade IIIb	5	7	
Grade IVa	3	6	
No.complication			0.35
Yes	37	29	
No	36	43	
No.90.days.death			0.60
Yes	72	3	
No	1	69	
No.90.days.return			1.00
Yes	66	66	
No	7	6	
No.Blood.transfusion			0.68
Yes	53	49	
No	20	23	
R0.resection			0.41
Yes	67	62	
No	6	10	
Textbook.outcome			0.40
Yes	26	20	
No	47	52	

Comparison of postoperative outcomes and complications between the high SII and low SII groups.

### 3.2 Patient grouping

The optimal cutoff value for SII was determined using ROC curve analysis based on overall survival (OS) and disease-free survival (DFS). The “surv_cutpoint” function in R was employed to identify the threshold that maximized the log-rank statistic, ensuring the strongest separation between high and low SII groups. This approach yielded an optimal SII cutoff value of 672.8, which was used to stratify patients into High SII and Low SII groups. Patients were categorized into high SII (n=65) and low SII (n=80) groups based on the median SII value of 672.8. Most baseline characteristics, including demographics, liver function tests, and tumor features, were comparable between groups. However, patients in the high SII group exhibited significantly higher CA19-9 levels compared to the low SII group (p=0.04). Based on this finding, patients were further stratified into four subgroups: Low SII & Low CA19-9 (n=36), Low SII & High CA19-9 (n=37), High SII & Low CA19-9 (n=36), and High SII & High CA19-9 (n=36).

### 3.3 Associations between SII and overall survival (OS)

The association between systemic immune-inflammation index (SII) and overall survival (OS) was assessed using Kaplan-Meier survival analysis. Patients were divided into two groups based on the median SII value (672.8): a high SII group (n = 65) and a low SII group (n = 80). The survival curves demonstrated a significant difference in OS between the two groups (p = 0.0027), with patients in the low SII group showing a markedly better survival probability compared to those in the high SII group.

The number of patients at risk decreased progressively over the follow-up period in both groups. At 1,000 days post-surgery, the survival probability remained substantially higher in the low SII group compared to the high SII group. By 3,000 days, only a few patients from either group remained at risk, but the trend favoring the low SII group persisted throughout the observation period.

**Figure 2:**
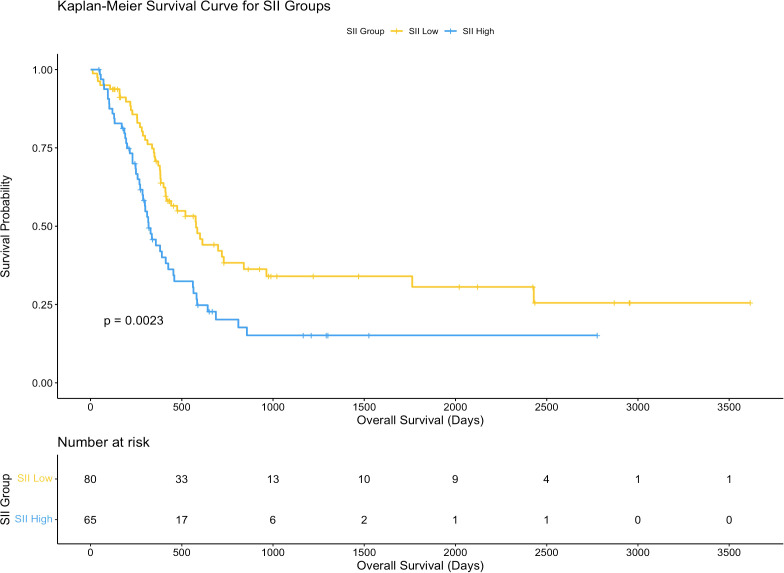
Kaplan-Meier curves for overall survival based on SII. Survival curves comparing overall survival (OS) between high and low systemic immune-inflammation index (SII) groups.

These results suggest that a higher SII is associated with worse overall survival in hilar cholangiocarcinoma patients receiving post-surgery adjuvant therapy. This indicates that systemic inflammation, as reflected by the SII, may play a significant role in influencing long-term outcomes in this patient population. Further details are depicted in **Figure 2**.

### 3.4 Associations between SII and disease-free survival (DFS)

The association between CA19-9 levels and overall survival (OS) was assessed using Kaplan-Meier survival analysis. Patients were divided into two groups based on CA19-9 levels (high vs. low). The Kaplan-Meier survival curves did not show a statistically significant difference in OS between the two groups (p = 0.072).

Further analysis revealed that the distribution of CA19-9 levels differed between the high SII and low SII groups. Patients in the high SII group exhibited a broader range and higher median CA19-9 levels compared to those in the low SII group. This distribution pattern suggests a potential link between systemic inflammation (as indicated by SII) and tumor burden (as reflected by CA19-9 levels). However, despite the differences in CA19-9 distribution between the SII groups, CA19-9 levels alone were not sufficient to independently predict OS in hilar cholangiocarcinoma patients.

**Figure 3:**
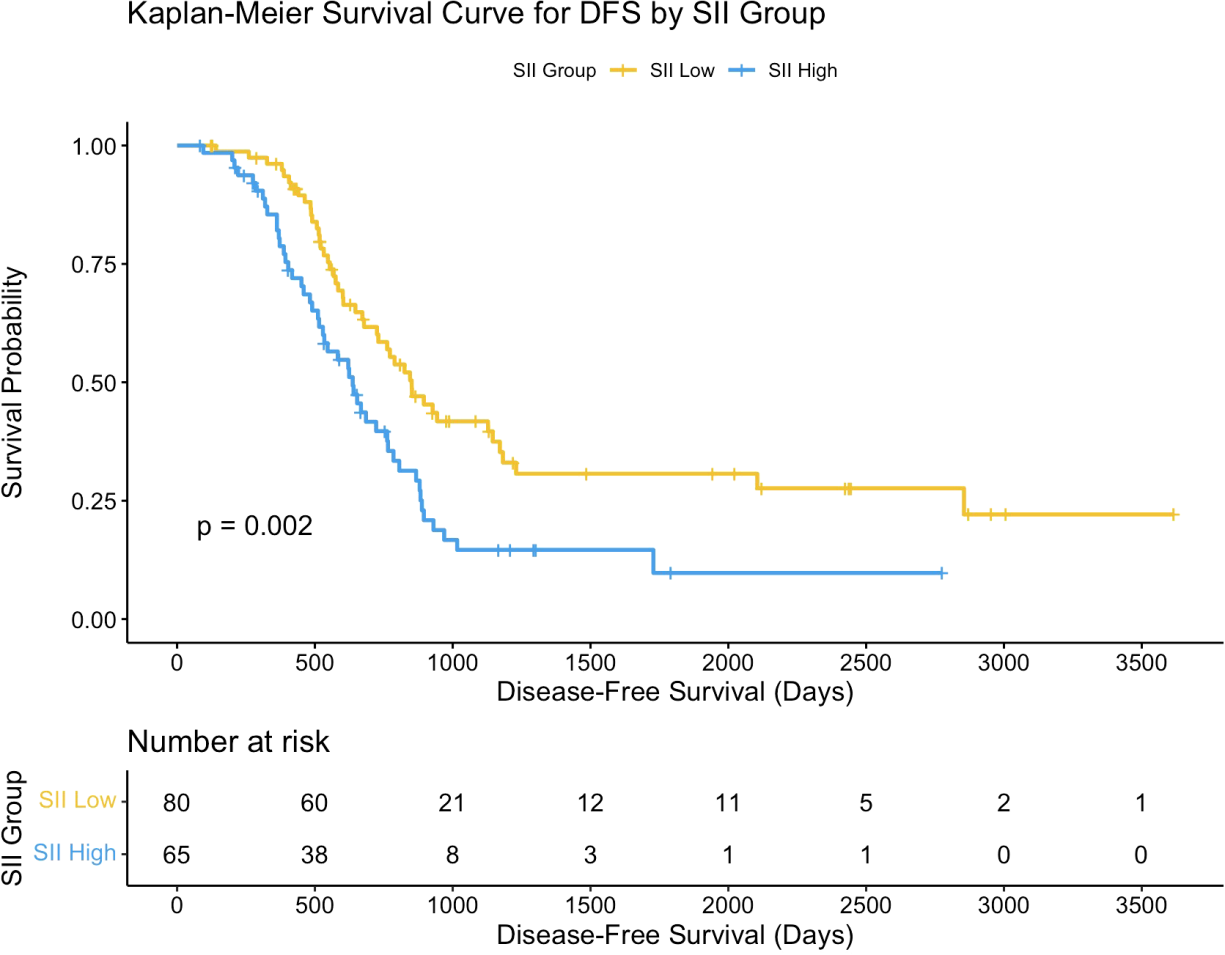
Kaplan-Meier curves for overall survival based on CA19-9. Survival curves comparing overall survival (OS) between high and low CA19-9 groups.

These findings highlight the complex interplay between systemic inflammation and tumor burden in influencing survival outcomes. While CA19-9 is a valuable tumor marker, its prognostic significance may be limited unless considered in conjunction with other factors, such as systemic immune-inflammatory status. Further details are depicted in **Figure 3**.

### 3.5 Association between CA19-9 level and survival outcomes

**Figure 4:**
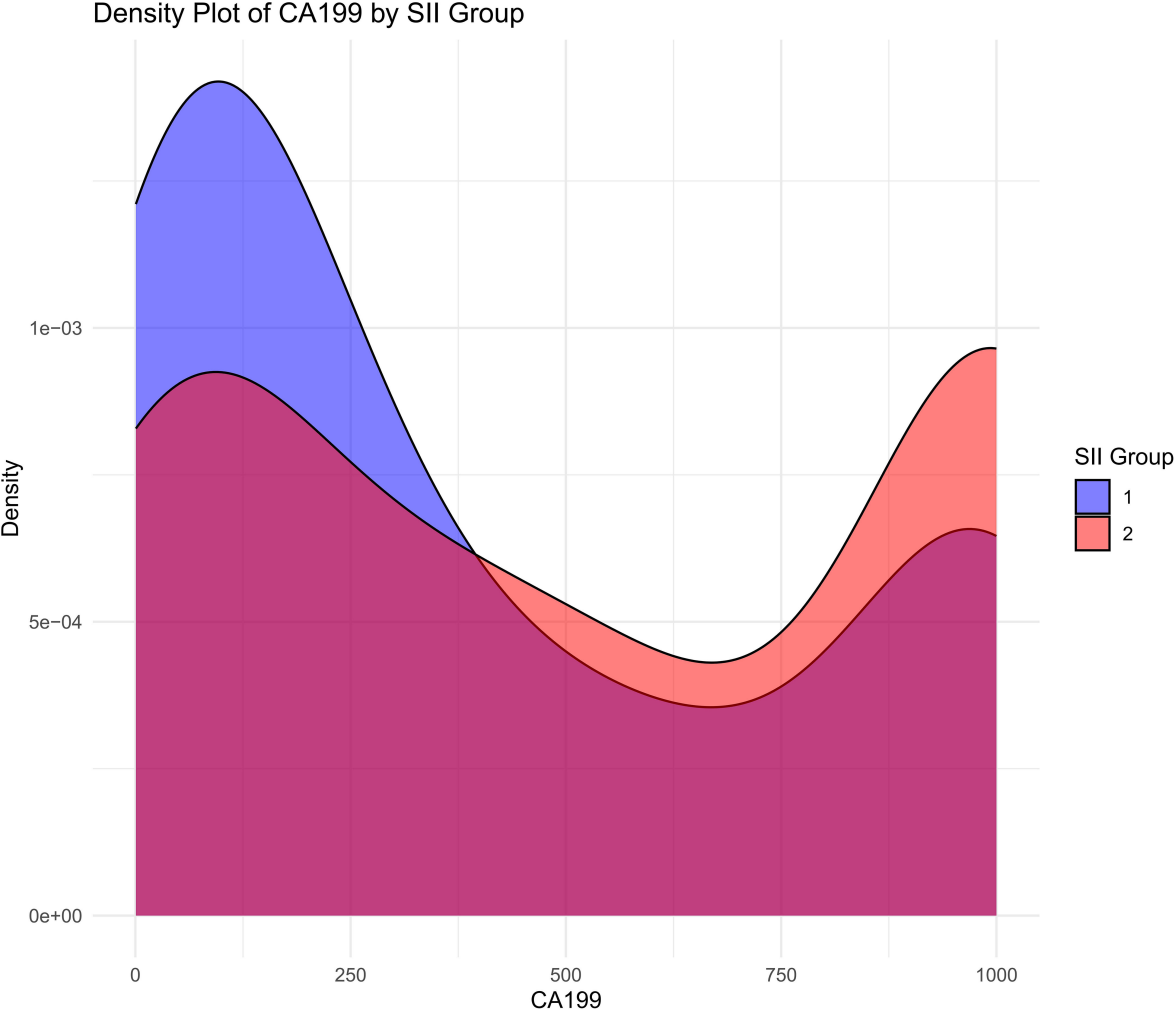
Distribution of CA19-9 levels by SII groups. Density plot showing the distribution of CA19-9 levels in high SII and low SII groups.

**Figure 5:**
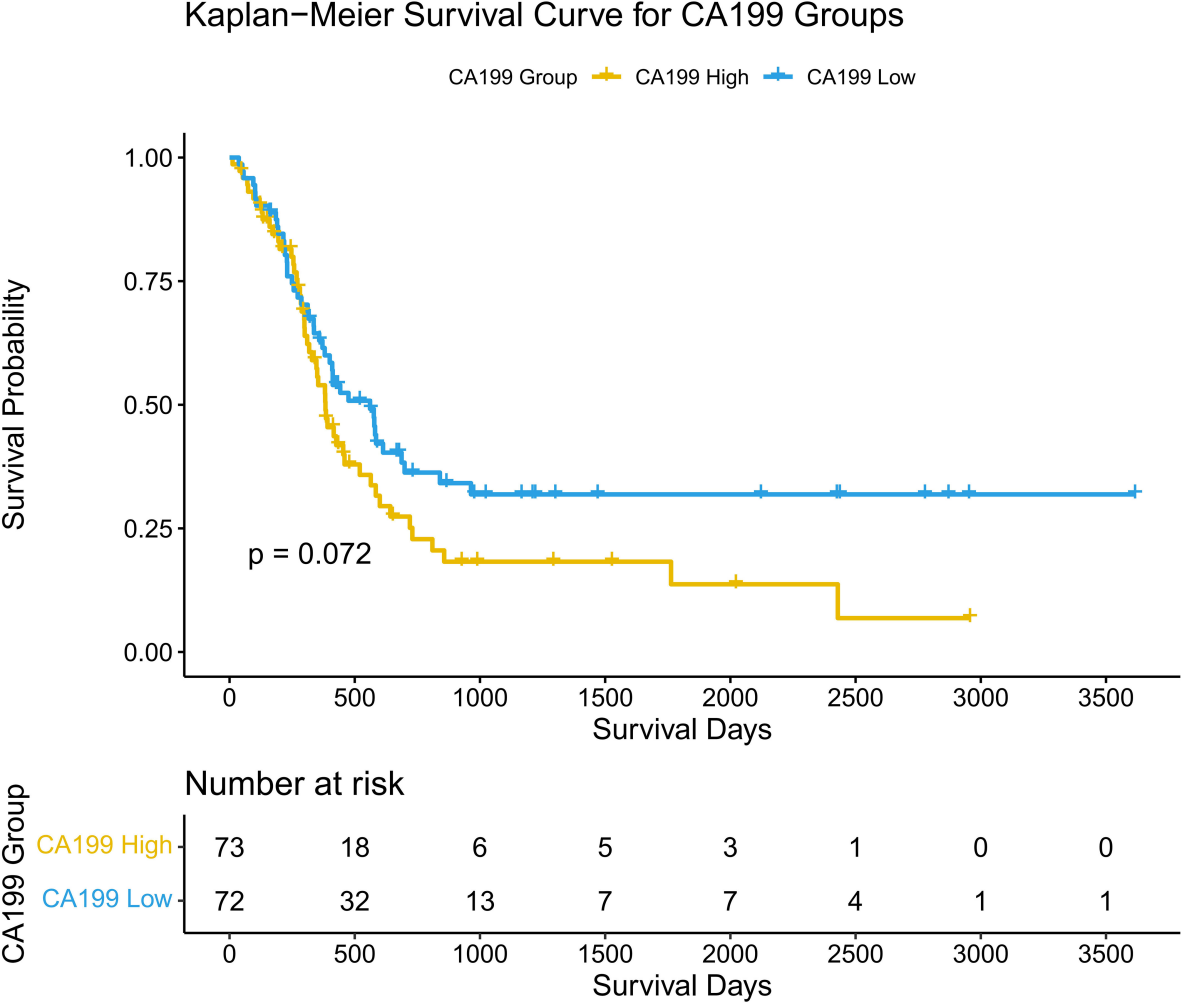
Kaplan-Meier curves for disease-free survival based on CA19-9. Survival curves comparing disease-free survival (DFS) between high and low CA19-9 groups.

The density plot illustrates the distribution of CA19-9 levels across the high-SII and low-SII groups, showing that CA19-9 levels are generally higher in the high-SII group compared to the low-SII group (**Figure 4**). The Kaplan-Meier survival curve further demonstrates the prognostic value of CA19-9, with patients in the low-CA19-9 group showing better overall survival probabilities than those in the high-CA19-9 group; however, the difference did not reach statistical significance (p = 0.072) (**Figure 5**). These findings suggest that higher CA19-9 levels may be associated with worse survival outcomes, potentially reflecting the aggressive tumor phenotype and its interplay with systemic inflammation, as indicated by SII.

### 3.6 Associations between SII, CA19-9, and overall survival (OS)

**Figure 6:**
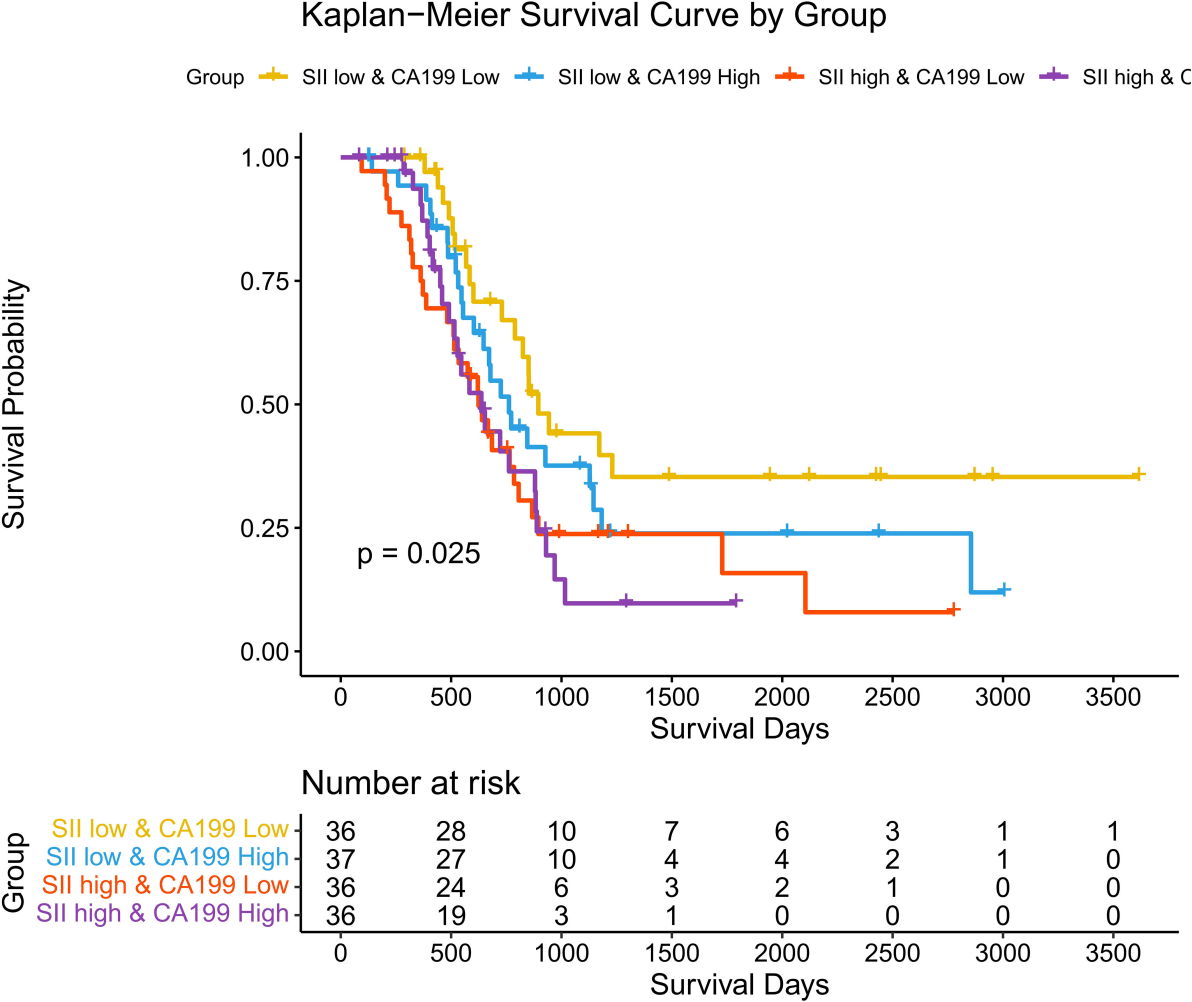
Kaplan-Meier curves for overall survival stratified by SII and CA19-9. Survival curves showing overall survival (OS) across four groups stratified by SII and CA19-9 levels.

Associations between SII, CA19-9, and Overall Survival (OS): To investigate the combined impact of systemic immune-inflammation index (SII) and tumor marker CA19-9 on overall survival (OS), patients were stratified into four groups: SII low & CA19-9 low, SII low & CA19-9 high, SII high & CA19-9 low, and SII high & CA19-9 high. Kaplan-Meier survival analysis revealed significant differences in OS among the four groups (p = 0.025). Patients in the SII low & CA19-9 low group demonstrated the most favorable survival outcomes, serving as the reference group with a hazard ratio (HR) of 1.00. The hazard ratios for the other groups highlight the increased risk associated with elevated SII and CA19-9 levels. Specifically, the SII low & CA19-9 high group had an HR of 1.51 (95% CI: 0.82–2.79, p = 0.1856), indicating a trend toward worse outcomes, although not statistically significant. The SII high & CA19-9 low group showed a significantly increased HR of 2.19 (95% CI: 1.21–3.97, p = 0.0101), emphasizing the adverse impact of elevated SII. The SII high & CA19-9 high group had the highest HR at 2.29 (95% CI: 1.23–4.25, p = 0.0088), demonstrating the combined negative effects of systemic inflammation and tumor burden on survival. These findings underscore the interplay between systemic inflammation and tumor marker CA19-9 in shaping long-term survival outcomes. Patients with both high SII and high CA19-9 levels are at the greatest risk of mortality, while low SII and CA19-9 levels predict the best prognosis. This highlights the importance of considering both inflammatory and tumor-related markers when evaluating prognosis in hilar cholangiocarcinoma patients. The above findings are detailed in **Figure 6** and **Table 2**.

**Table 2:** Combined impact of SII and CA19-9 on overall survival.

Group	HR	CI_lower	CI_upper	p_value
groupSII low & CA199 low	1	/	/	/
groupSII low & CA199 High	1.51	0.82	2.79	0.1856
groupSII high & CA199 Low	2.19	1.21	3.97	0.0101
groupSII high & CA199 High	2.29	1.23	4.25	0.0088

Associations between systemic immune-inflammation index (SII), CA19-9 levels, and overall survival (OS).

### 3.7 Associations between SII, CA19-9, and disease-free survival (DFS)

**Figure 7:**
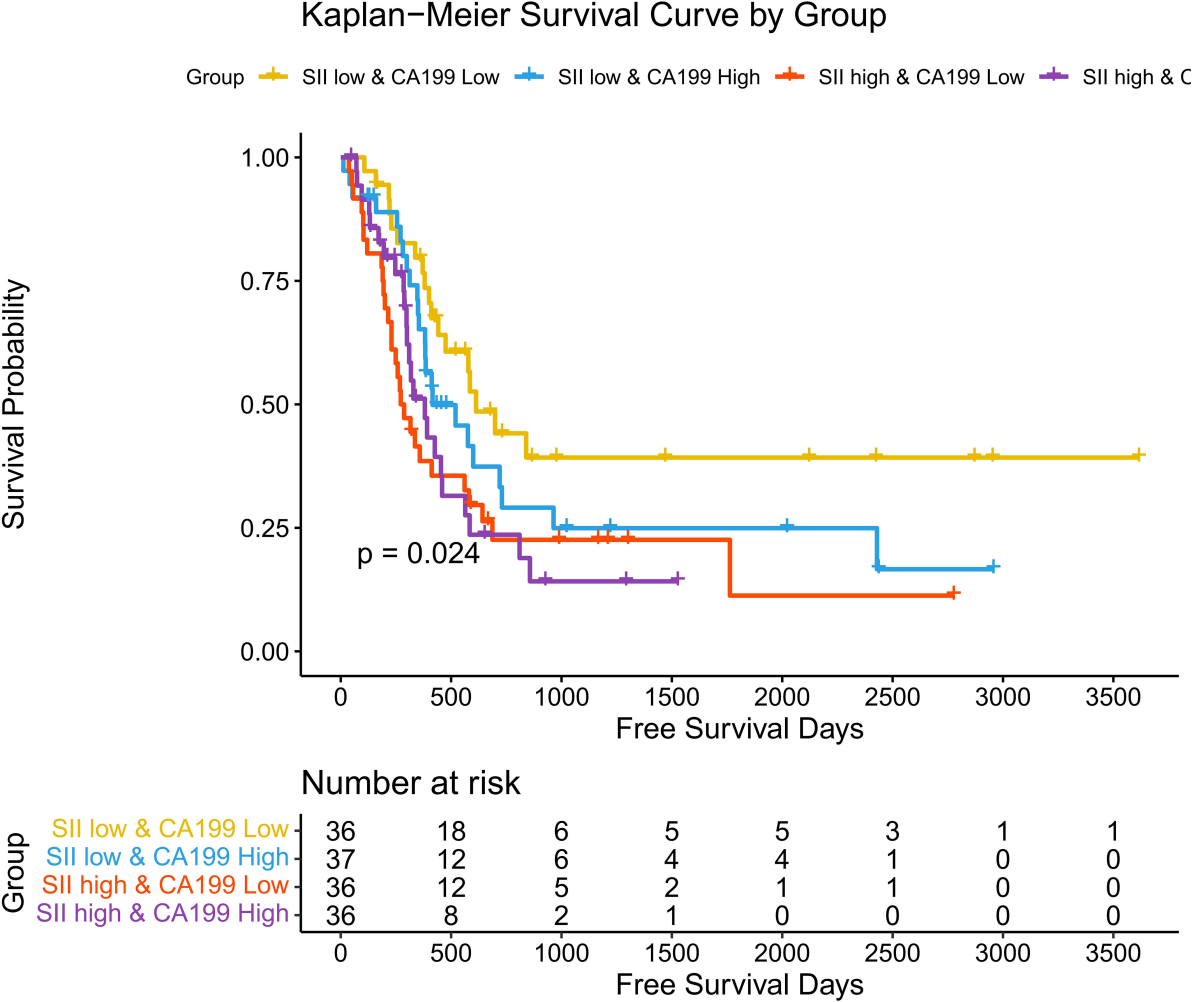
Hazard ratios for disease-free survival stratified by SII and CA19-9. Forest plot showing hazard ratios (HRs) for disease-free survival (DFS) across four groups stratified by SII and CA19-9 levels.

Associations between SII, CA19-9, and Disease-Free Survival (DFS): A similar trend was observed for disease-free survival (DFS) as with overall survival (OS). Kaplan-Meier survival analysis demonstrated significant differences in DFS among the four stratified groups (p = 0.024). Patients in the SII low & CA19-9 low group exhibited the most favorable DFS, maintaining the highest probability of being disease-free over the follow-up period. Conversely, patients in the SII high & CA19-9 high group had the shortest DFS, with a rapid decline in survival probability. The intermediate groups followed the same trend observed in OS, with the SII low & CA19-9 high group achieving slightly better DFS than the SII high & CA19-9 low group, underscoring the negative impact of systemic inflammation on survival. Hazard ratios (HR) further highlight the disparities among these groups. The SII low & CA19-9 low group served as the reference (HR = 1.00). The SII low & CA19-9 high group had an HR of 1.53 (95% CI: 0.83–2.82, p = 0.1746), indicating a trend toward worse outcomes that did not reach statistical significance. The SII high & CA19-9 low group showed a significantly increased HR of 2.29 (95% CI: 1.26–4.15, p = 0.0062), while the SII high & CA19-9 high group also had a significantly elevated HR of 2.16 (95% CI: 1.17–3.99, p = 0.0144). These findings emphasize the combined adverse effects of systemic inflammation and elevated tumor burden on DFS, with high SII and high CA19-9 levels conferring the greatest risk of disease recurrence. The above findings are detailed in **Figure 7** and **Table 3**. A *post-hoc* power calculation was performed using the Schoenfeld formula for Cox proportional hazards models, based on the observed hazard ratios, event rates, and sample size. For the primary analysis comparing high and low SII groups, the study achieved 85% power (α=0.05, HR=2.19, 98 events). Subgroup analyses (e.g., SII + CA19-9 stratification) had relatively lower power (72%, HR=2.29, 52 events), reflecting the exploratory nature of these findings. Power calculations were conducted using the R package powerSurvEpi (version 1.1-3).

**Table 3:** Combined impact of SII and CA19-9 on disease-free survival.

Group	HR	CI_lower	CI_upper	p_value
groupSII low & CA199 Low	1	/	/	/
groupSII low & CA199 High	1.53	0.83	2.82	0.1746
groupSII high & CA199 Low	2.29	1.26	4.15	0.0062
groupSII high & CA199 High	2.16	1.17	3.99	0.0144

Associations between systemic immune-inflammation index (SII), CA19-9 levels, and disease-free survival (DFS).

### 3.8 Prognostic factors for included patinets

This study identified several prognostic factors significantly associated with OS in hilar cholangiocarcinoma (HCCA). Elevated lymph node metastasis (HR = 1.72, p = 0.0084), local organ metastasis (HR = 3.22, p = 0.0001), and intrahepatic metastasis (HR = 2.04, p = 0.0025) were strongly linked to worse survival outcomes, underscoring the impact of metastatic spread on patient prognosis. Additionally, systemic inflammatory and nutritional markers, such as albumin levels (HR = 0.63, p = 0.0414), were inversely correlated with survival, reflecting the role of systemic health in influencing outcomes. Technical and pathological factors also played a role, with R0 resection (HR = 0.53, p = 0.0393) and the absence of blood transfusion (HR = 0.63, p = 0.0329) being associated with better OS. Grade B/C postoperative bleeding (HR = 4.48, p = 0.0381) was a significant negative factor, highlighting the importance of managing perioperative complications. Furthermore, the tumor staging system, particularly N stage (HR = 1.62, p = 0.0189) and the comprehensive AJCC staging (HR = 1.31, p = 0.0086), demonstrated prognostic relevance, affirming their utility in risk stratification. The above findings are detailed in **Table 4A**.

**Table 4A:** Prognostic factors for overall survival.

	Univariate Analysis				Multivariate Analysis			
Variables	HR	95%CI_lower	95%CI_upper	p_value	HR	95%CI_lower	95%CI_upper	p_value
Hemoglobin	0.99	0.98	1	0.1945				
Total.Bilirubin	1.01	0.99	1.02	0.2433				
Direct.Bilirubin	1.01	0.99	1.03	0.2501				
Albumin	0.63	0.4	0.98	0.0414	0.98	0.93	1.03	0.5045
ALT	0.99	0.98	1.01	0.3928				
AST	0.99	0.97	1.02	0.5677				
Platelet	0.99	0.97	1.01	0.3786				
Prothrombin.Time	2.33	0.42	12.82	0.3315				
CEA	1.03	1	1.07	0.0414	1	1	1.01	0.1308
CA125	1.02	0.98	1.05	0.4032				
biliary.drainage	1.05	0.68	1.6	0.8368				
Processed.Tumor.Size	1.09	0.98	1.21	0.0999	1.01	0.9	1.14	0.8517
Tumor.Type	1.06	0.69	1.63	0.7884				
Differentiation	1.09	0.74	1.61	0.6483				
Artery.invasion	0.91	0.58	1.41	0.6637				
Vein.invasion	1.15	0.72	1.85	0.5553				
Main.hepatic.vein.invasion	0.98	0.61	1.59	0.9477				
Vena.cava.invasion	0.75	0.3	1.85	0.5356				
Local.organ.metastasis	3.22	1.77	5.84	0.0001	3.12	1.59	6.12	0.0009
Intrahepatic.metastasis	2.04	1.28	3.24	0.0025	1.84	1.07	3.15	0.0271
Lymph.node.metastasis	1.72	1.15	2.59	0.0084	2.8	0.54	14.41	0.2177
hospital.stay	1	0.99	1.02	0.8185				
hospital.mortality	_	_	_	_				
Reoperation	0.71	0.22	2.24	0.5544				
Grade.A.liver.failure	0.65	0.16	2.66	0.5541				
Grade.B.C.liver.failure	1.24	0.3	5.08	0.7646				
Grade.A.bile.leakage	1.4	0.76	2.58	0.2797				
Grade.B.C.bile.leakage	0.46	0.06	3.32	0.443				
Grade.A.postoperative.bleeding	0.34	0.08	1.37	0.1284				
Grade.B.C.postoperative.bleeding	4.48	1.09	18.46	0.0381	4.46	0.96	20.78	0.057
Clavien.Dindo.grade	0.98	0.83	1.16	0.8271				
No.complication	0.98	0.65	1.47	0.9245				
No.90.days.death	0.55	0.17	1.76	0.3151				
No.90.days.return	0.77	0.37	1.6	0.49				
No.Blood.transfusion	0.63	0.42	0.96	0.0329	0.96	0.59	1.57	0.8853
R0.resection	0.53	0.28	0.97	0.0393	0.51	0.27	0.98	0.0489
textbook.outcome	0.75	0.48	1.16	0.2013				
TSTAGE	1.26	0.99	1.61	0.0584	1.03	0.69	1.53	0.8785
NSTAGE	1.62	1.08	2.44	0.0189	0.36	0.07	1.82	0.2159
MSTAGE	_	_	_	_				
AJCC	1.31	1.07	1.6	0.0086	1.28	0.78	2.09	0.3289

Multivariate analysis of prognostic factors associated with overall survival in hilar cholangiocarcinoma patients.

This study identified several significant prognostic factors associated with DFS in hilar cholangiocarcinoma (HCCA). Elevated lymph node metastasis (HR = 1.55, p = 0.0341), local organ metastasis (HR = 2.22, p = 0.0083), and intrahepatic metastasis (HR = 1.66, p = 0.0315) were strongly linked to a higher risk of recurrence, emphasizing the impact of metastatic spread on disease progression. Among tumor staging systems, the AJCC staging (HR = 1.24, p = 0.0336) was significantly associated with DFS, confirming its value in stratifying recurrence risk. Furthermore, elevated carcinoembryonic antigen (CEA) levels (HR = 1.05, p = 0.0272) emerged as a predictive factor for poor DFS, highlighting its role as a biomarker of tumor aggressiveness. These findings suggest that both tumor burden and the extent of metastasis critically influence recurrence-free survival in HCCA patients. The above findings are detailed in **Table 4B**.

**Table 4B:** Prognostic factors for disease-free survival.

	Univariate Analysis				Multivariate Analysis			
Variables	HR	95%CI_lower	95%CI_upper	p_value	HR	95%CI_lower	95%CI_upper	p_value
Hemoglobin	0.99	0.98	1	0.2762				
Total.Bilirubin	1.01	0.99	1.02	0.3629				
Direct.Bilirubin	1.01	0.99	1.03	0.3489				
Albumin	0.7	0.44	1.1	0.1223				
ALT	1	0.98	1.01	0.5995				
AST	1	0.98	1.02	0.9047				
Platelet	0.98	0.96	1.01	0.1799				
Prothrombin.Time	1.57	0.3	8.09	0.5894				
CEA	1.05	1.01	1.09	0.0272	1	1	1.01	0.0295
CA125	1	0.97	1.04	0.9154				
biliary.drainage	1.01	0.66	1.55	0.9508				
Processed.Tumor.Size	1.05	0.95	1.17	0.329				
Tumor.Type	1.36	0.89	2.09	0.1603				
Differentiation	1	0.67	1.5	0.9897				
Artery.invasion	0.89	0.57	1.39	0.6148				
Vein.invasion	1.15	0.72	1.85	0.5538				
Main.hepatic.vein.invasion	0.84	0.52	1.35	0.4737				
Vena.cava.invasion	0.83	0.34	2.05	0.6887				
Local.organ.metastasis	2.22	1.23	4.02	0.0083	2.12	1.13	3.98	0.0195
Intrahepatic.metastasis	1.66	1.05	2.62	0.0315	1.53	0.93	2.51	0.0934
Lymph.node.metastasis	1.55	1.03	2.32	0.0341	4.32	0.85	21.94	0.078
hospital.stay	1	0.98	1.02	0.9255				
hospital.mortality	_	_	_	_				
Reoperation	0.73	0.23	2.3	0.5892				
Grade.A.liver.failure	0.85	0.21	3.46	0.8212				
Grade.B.C.liver.failure	0.98	0.24	4.01	0.9825				
Grade.A.bile.leakage	1.47	0.8	2.7	0.212				
Grade.B.C.bile.leakage	0.49	0.07	3.49	0.4727				
Grade.A.postoperative.bleeding	0.33	0.08	1.35	0.1236				
Grade.B.C.postoperative.bleeding	3.47	0.85	14.24	0.0838	3.31	0.74	14.93	0.1186
Clavien.Dindo.grade	0.97	0.83	1.14	0.7316				
No.complication	1	0.66	1.49	0.9829				
No.90.days.death	0.9	0.28	2.84	0.8539				
No.90.days.return	0.68	0.33	1.41	0.3047				
No.Blood.transfusion	0.69	0.45	1.05	0.0847	0.84	0.54	1.29	0.4211
R0.resection	0.54	0.3	1	0.05	0.5	0.26	0.97	0.041
textbook.outcome	0.78	0.5	1.2	0.2561				
TSTAGE	1.18	0.93	1.5	0.1704				
NSTAGE	1.45	0.96	2.17	0.0747	0.21	0.05	0.97	0.0461
MSTAGE	_	_	_	_				
AJCC	1.24	1.02	1.51	0.0336	1.27	0.89	1.8	0.1812

Multivariate analysis of prognostic factors associated with disease-free survival in hilar cholangiocarcinoma patients.

## 4 Discussions

This study systematically evaluated the prognostic value of the systemic immune-inflammation index (SII) and carbohydrate antigen 19-9 (CA19-9) in patients with hilar cholangiocarcinoma (HCCA) receiving post-surgery adjuvant therapy. While elevated levels of both SII and CA19-9 were associated with worse survival outcomes, their combined assessment offers superior prognostic accuracy. Specifically, integrating SII and CA19-9 into a predictive model demonstrated enhanced ability to stratify patients based on their recurrence and survival risks, supporting its potential as a robust tool for therapeutic decision-making.

The SII, derived from platelet, neutrophil, and lymphocyte counts, reflects systemic inflammation and its role in tumor progression and therapy resistance (17). Elevated SII signifies increased neutrophil and platelet activity alongside lymphocytopenia, creating an immunosuppressive microenvironment conducive to tumor progression (3). This has been previously demonstrated in hepatocellular carcinoma, colorectal cancer, and other malignancies, where systemic inflammation impairs anti-tumor immunity and drives disease progression. Consistent with these findings, our study confirms that HCCA patients with high SII levels have significantly worse overall survival (OS) and disease-free survival (DFS) compared to those with lower SII values. These results emphasize the detrimental impact of systemic inflammation on the tumor microenvironment and immune surveillance (18, 19).

Notably, elevated SII levels, reflecting systemic inflammation, are associated with reduced efficacy of adjuvant therapy in multiple malignancies, likely due to an immunosuppressive microenvironment (20, 21). Our findings suggest a similar trend in HCCA, where patients with high SII levels showed poorer survival outcomes despite receiving adjuvant therapy. Mechanistically, systemic inflammation may impair the immune-mediated tumor-killing effects of chemotherapy and reduce the efficacy of radiotherapy by promoting angiogenesis and hypoxia within the tumor microenvironment (22, 23). Conversely, patients with low SII levels may benefit more from adjuvant therapy due to a more favorable immune status that enhances treatment responsiveness (24). This underscores the need for incorporating SII into treatment planning to identify patients who may require additional therapeutic strategies to counteract the effects of systemic inflammation.

CA19-9, a widely recognized tumor marker in biliary tract cancers, provides complementary information to SII by reflecting tumor burden and biological aggressiveness (22). Elevated CA19-9 levels in our study were associated with key pathological features such as vascular invasion, lymph node metastasis, and perineural invasion, all of which significantly impair survival outcomes (21). Although CA19-9 was not independently associated with OS in our cohort (p > 0.05), it remained a significant predictor of DFS, highlighting its relevance in identifying patients at high risk of recurrence. Mechanistically, CA19-9-positive tumors often exhibit glycolytic reprogramming driven by KRAS mutations and hypoxia-inducible factor 1-alpha (HIF-1α) activation, which further supports tumor growth, immune evasion, and metastasis (23, 25).Our findings highlight a critical synergy between systemic inflammation (SII) and tumor burden (CA19-9) in determining survival outcomes. While CA19-9 alone showed limited prognostic value for OS, its combination with SII revealed a context-dependent relationship where systemic inflammation amplifies the adverse effects of tumor biology. Mechanistically, systemic inflammation fosters an immunosuppressive microenvironment through neutrophilia and lymphopenia, promoting tumor progression and metastasis. Concurrently, CA19-9, a marker of aggressive tumor behavior, may reflect glycolytic reprogramming and immune evasion. This bidirectional interaction is supported by studies in other malignancies, where neutrophil extracellular traps (NETs) enhance metastasis by facilitating tumor cell adhesion, and platelet-tumor cell interactions shield circulating tumor cells from immune clearance. Clinically, this synergy underscores the need for dual therapeutic strategies targeting both inflammation (e.g., anti-IL-6 agents) and tumor metabolism (e.g., glycolysis inhibitors). Future studies should explore whether modulating systemic inflammation improves outcomes in patients with elevated CA19-9.

The combined assessment of SII and CA19-9 offers a more comprehensive understanding of tumor biology and patient prognosis. While CA19-9 reflects tumor-specific factors such as metabolic activity and aggressiveness, SII captures the systemic inflammatory status of the host (19). Patients with both high SII and elevated CA19-9 levels demonstrated the poorest DFS, emphasizing the synergistic impact of systemic inflammation and tumor metabolism on recurrence and progression. This combination is particularly valuable for identifying high-risk patients who may require intensified adjuvant therapy or novel therapeutic approaches targeting both systemic inflammation and tumor biology (26). Additionally, the interplay between systemic inflammation (SII) and tumor burden (CA19-9) is underpinned by several biological mechanisms that collectively promote tumor progression and metastasis. Elevated SII, characterized by neutrophilia, thrombocytosis, and lymphopenia, creates an immunosuppressive microenvironment that facilitates tumor immune evasion and metastatic spread. Neutrophil extracellular traps (NETs), for instance, have been shown to promote metastasis by enhancing the adhesion of circulating tumor cells to distant organs, a process that may be further amplified in CA19-9-positive tumors due to their enhanced glycolytic activity and invasive potential (27). Platelets, another key component of systemic inflammation, play a dual role in promoting metastasis. They shield circulating tumor cells from immune surveillance and facilitate their extravasation into distant tissues. Platelet-derived TGF-β and P-selectin have been implicated in enhancing the epithelial-mesenchymal transition (EMT) and metastatic niche formation, particularly in tumors with high CA19-9 expression (28). Moreover, systemic inflammation drives metabolic reprogramming in tumors, favoring glycolysis and lactate production—a hallmark of CA19-9-positive tumors. Inflammatory cytokines such as IL-6 and TNF-α upregulate HIF-1α and KRAS signaling, further enhancing tumor aggressiveness and resistance to therapy (29). Together, these mechanisms provide a plausible explanation for the synergistic effect of high SII and high CA19-9 on poor survival outcomes.

From a clinical perspective, integrating SII and CA19-9 into prognostic models can improve post-surgical risk stratification and guide therapeutic decisions in HCCA (30). Patients with elevated levels of both markers may benefit from closer surveillance and tailored adjuvant therapy regimens that address both inflammation and tumor-specific pathways (31). Emerging therapies, such as anti-inflammatory agents targeting neutrophil and platelet activity or inhibitors of glycolysis and HIF-1α signaling, could be particularly beneficial for this high-risk population (32). Furthermore, combining these biomarkers with additional molecular and clinical factors, such as KRAS mutation status, could further enhance predictive accuracy and inform personalized treatment strategies (33).

However, this study has limitations. Our study has several limitations. First, as a retrospective single-center analysis, it may be subject to selection bias and limited generalizability. Second, treatment heterogeneity in adjuvant therapies (chemotherapy, radiotherapy, or combinations) may introduce residual confounding, despite adjustment in multivariate models. Third, the timing of SII and CA19-9 measurements (preoperative only) may not capture dynamic changes post-surgery or during adjuvant therapy. Postoperative inflammation or biliary drainage could alter these markers, potentially affecting their prognostic value. Future studies should evaluate serial measurements to better understand their clinical utility. Finally, the retrospective design precludes causal inferences. Prospective multicenter studies with standardized protocols are needed to validate our findings and enhance generalizability. Despite these limitations, our study provides valuable insights into the prognostic role of SII and CA19-9 in HCCA, supporting their integration into risk stratification and therapeutic decision-making. Further mechanistic investigations are warranted to elucidate the interplay between systemic inflammation, tumor metabolism, and molecular alterations, which may reveal novel therapeutic targets.

## 5 Conclusions

In, this study highlights the prognostic significance of integrating SII and CA19-9 in patients with HCCA undergoing post-surgery adjuvant therapy. While SII reflects systemic inflammation and CA19-9 captures tumor aggressiveness, their combined use provides a more accurate risk stratification and offers insights into personalized treatment strategies. Understanding the interaction between systemic inflammation and tumor biology may pave the way for novel therapeutic approaches aimed at improving survival outcomes in this high-risk patient population.

## Data Availability

The original contributions presented in the study are included in the article/supplementary material. Further inquiries can be directed to the corresponding authors.
